# Cytokine Signatures for Lung Cancer Diagnosis in African American Populations

**DOI:** 10.3390/jpm14010117

**Published:** 2024-01-20

**Authors:** Qixin Leng, Pushpa Dhilipkannah, Feng Jiang

**Affiliations:** Departments of Pathology, University of Maryland School of Medicine, Baltimore, MD 21201, USA

**Keywords:** biomarkers, cytokines, plasma, lung cancer, African Americans

## Abstract

**Simple Summary:**

Lung cancer is the primary cancer fatality, disproportionately affecting African Americans (AAs), who experience higher incidence and mortality rates than other ethnic groups. Cytokines play crucial roles in cancer development. Eight key cytokines were scrutinized in 104 lung cancer patients and 48 controls. The levels of IL-8, IFN-γ, and TNF-α were elevated in both AA and White American (WA) lung cancer cases. IL-10 and MCP-1 exhibited pronounced elevation specifically in AA lung cancer patients, with MCP-1 being associated with lung adenocarcinoma. IL-6 levels were specifically elevated in WA lung cancer patients and associated with lung adenocarcinoma. The combined use of specific cytokines showed promise in diagnosing lung cancer, with IL-8, IL-10, and MCP-1 achieving 76% sensitivity and 79% specificity in AAs, and IL-6 and IL-8 combined offering 76% sensitivity and 74% specificity in WAs. These diagnostic biomarkers were validated in an independent cohort of 58 cases and 58 controls. These ethnicity-related cytokine biomarkers hold potential for early detection and addressing disparities in lung cancer outcomes.

**Abstract:**

Lung cancer is the leading cause of cancer-related deaths among both men and women. African Americans (AAs) experience disproportionately higher incidence and mortality compared to other ethnic groups. Cytokines play multifaceted and crucial roles in the initiation, progression, and spread of cancer. Our aim was to identify cytokine biomarkers for the early detection of lung cancer in AAs. We examined eight key cytokines (Interleukin-1, IL-6, IL-8, IL-10, IL-12p70, monocyte chemotactic protein-1 (MCP-1), interferon-gamma (IFN-γ), and tumor necrosis factor-alpha (TNF-α)) in the plasma of 104 lung cancer patients and 48 cancer-free individuals using the FirePlex Immunoassay. These findings were subsequently validated in a separate cohort of 58 cases and 58 controls. IL-8, IFN-γ, and TNF-α exhibited elevated levels in both AA and White American (WA) lung cancer cases. Notably, IL-10 and MCP-1 displayed significant increases specifically in AA lung cancer patients, with MCP-1 levels associated with lung adenocarcinoma cases. Conversely, WA lung cancer patients showed heightened IL-6 levels, particularly linked to lung adenocarcinoma. The combined use of specific cytokines showed promise in lung cancer diagnosis, with IL-8, IL-10, and MCP-1 achieving 76% sensitivity and 79% specificity in AAs and IL-6 and IL-8 combined offering 76% sensitivity and 74% specificity in WAs. These diagnostic biomarkers were validated in the independent cohort. The ethnicity-related cytokine biomarkers hold promise for diagnosing lung cancer in AAs and WAs, potentially addressing the observed racial disparity.

## 1. Introduction

Lung cancer consistently ranks as the leading cause of cancer-related mortality for both men and women in the USA [1]. A total of 85% of all lung cancer cases are categorized as non-small-cell lung cancer (NSCLC). NSCLC primarily manifests as one of two major histological subtypes: adenocarcinoma (AC) and squamous cell carcinoma (SCC) [1]. Early detection can markedly reduce mortality associated with NSCLC [1]. However, current diagnostic methods are insufficient in enabling the early detection of lung cancer. Furthermore, discernible disparities in NSCLC incidence and outcomes exist among diverse ethnicities, with African Americans (AAs) experiencing both a higher prevalence and higher mortality rates from the disease [2]. The disparities are not solely sociodemographic but also biological, involving variations in tumor biology, genetics, and molecular alterations [3]. Specific examples of genetic disparities in lung cancer susceptibility among AAs include the identification of loci on chromosomes 5p15 and 15q25 [4]. Additionally, disparities have been observed in the methylation levels of functionally relevant genes, such as those in the nuclear receptor subfamily 3 [5]. Moreover, AAs demonstrate a higher prevalence of epidermal growth factor receptor (EGFR) mutations [6], and they are less susceptible to the alternative polyadenylation of mRNA transcripts in lung cancer compared to WAs [7]. Such molecular and genetic discrepancies are pivotal to lung cancer disparities and proffer invaluable insights into potential biomarkers for NSCLC in AAs [8].

Chronic inflammation and immune responses are integral to lung tumorigenesis [9]. Cytokines are produced by either surrounding or malignant cells as part of the inflammatory process and the hypoxic conditions [9]. Cytokines can pervade tumor cell growth, invasion, and metastasis. For instance, elevated serum levels of IL-6 and IL-8 have been correlated with reduced survival durations in lung cancer patients [10]. Interestingly, elevated levels of specific cytokines are linked to an increased risk of lung cancer within the AA population [11,12]. Accordingly, we posit that via analyzing cytokines in the plasma of NSCLC patients across both AA and WA cohorts, we can develop noninvasive lung cancer biomarkers that are individually tailored to specific ethnicities.

## 2. Materials and Methods

### 2.1. Patients and Clinical Information

Our research received approval from the Institutional Review Board of the University of Maryland Baltimore. This study received approval from the Institutional Review Board of the University of Maryland Baltimore (IRB HP-00040666). We received consent from and recruited both lung cancer patients and cancer-free smokers. Specifically, smokers aged 50–80, possessing a minimum of a 20-pack-per-year smoking history, and being either current or former smokers (having quit within the last 15 years) were recruited. Exclusion criteria encompassed the following: age < 21 years, pregnancy or lactation, current pulmonary infection, thoracic surgery within the preceding 6 months, and radiotherapy to the chest within the past year. Demographic, radiological, and clinical variables were obtained via a comprehensive review of medical records. Surgical pathologic staging was performed in adherence with the TNM classification guidelines outlined by the International Union Against Cancer, the American Joint Committee on Cancer, and the International Staging System for Lung Cancer. Furthermore, histopathologic classification adhered to the guidelines published by the World Health Organization. In the present study, we enrolled 104 NSCLC patients and 48 cancer-free smokers, comprising 45 AA lung cancer patients, 29 AA cancer-free smokers, 59 WA lung cancer patients, and 19 WA cancer-free smokers (Table 1). Among 104 NSCLC patients, there were 15 diagnosed with stage I, 32 with stage II, 23 with stage III, and 23 with stage IV. Additionally, there were 45 cases of adenocarcinoma (AC) and 34 cases of squamous cell carcinoma (SCC). The 48 cancer-free smokers had benign conditions: 19 had granulomatous inflammation, 18 exhibited nonspecific inflammatory changes, and 11 presented with lung infections. This cohort was used as an exploratory set. In the Baltimore VA Medical Center, we enrolled 58 NSCLC patients and 58 cancer-free smokers, comprising 28 AA lung cancer patients, 28 AA cancer-free smokers, 30 WA lung cancer patients, and 30 WA cancer-free smokers (Table 1). Among 58 NSCLC patients, there were 18 diagnosed with stage I, 16 with stage II, 14 with stage III, and 10 with stage IV. Additionally, there were 32 cases of AC and 26 cases of SCC. The 58 cancer-free smokers had benign conditions: 29 had granulomatous inflammations, 16 exhibited nonspecific inflammatory changes, and 13 presented with lung infections. In addition, to further validate whether cytokines can be used for the early detection of lung cancer in AAs, we acquired archived plasma samples from an additional cohort comprising 36 early-stage lung cancer patients within the AA population, alongside 38 cancer-free AAs (Supplementary Table S1).

### 2.2. Blood Collection and Plasma Preparation

Whole blood (10 mL) was collected from each participant using BD Vacutainer spray-coated K2EDTA Tubes (BD, Franklin Lakes, NJ, USA), adhering to the standard operating protocol delineated in our prior studies [13,14,15,16,17]. To ensure their integrity, specimens were processed within one hour of collection by centrifuging at 1000× *g* for 15 min at 4 °C. Subsequently, plasma was aliquoted, promptly frozen, and stored securely at −80 °C.

### 2.3. The Measurement of the Cytokines in Plasma

The FirePlex Immunoassay (Abcam, Cambridge, MA, USA) was utilized to measure eight crucial cytokines (IL-1, IL-6, IL-8, IL-10, IL-12p70, MCP-1, IFN-γ, and TNF-α), following the manufacturer’s guidelines. Initially, the 1× Capture Particle Solution was mixed thoroughly and dispensed into each well of the plate. After each application, vacuum filtration was used to remove buffers. Plasma samples, or predetermined standards, were then added to the wells and incubated for 1 h at room temperature with continuous shaking. This step was followed by the addition of the biotin detector antibody mix and subsequent washing to remove unbound material. Next, the 1× reporter solution was introduced, incubated, and subsequently washed away. Adding the run buffer marked the final step before data collection, which was performed using a flow cytometer calibrated with the appropriate run buffer. To ensure cytometer accuracy, the FirePlex Cytometer Setup Kit V2 was used, and actual immunoassay data acquisition was fine-tuned using two reference blank wells. Data were analyzed using the Fireplex Analysis Workbench (Abcam) by comparing sample readings against a standard curve created from known cytokine concentrations.

### 2.4. Statistical Analysis

We initially applied the Shapiro–Wilk Test to ascertain whether our data followed a normal distribution. In cases where the data were not normally distributed, we utilized the Mann–Whitney U Test to assess the statistical significance of cytokines between different groups. The relationship between cytokine levels and clinical and demographic data was evaluated using Spearman’s rank correlation coefficient. Feature selection was conducted using the least absolute shrinkage and selection operator (LASSO) in combination with logistic regression. To ensure a comprehensive and transparent statistical analysis, we adopted a multifaceted approach. We utilized 10-fold cross-validation, where the data were divided into 10 equal subsets, and the model was trained and tested across these subsets to evaluate its performance comprehensively. Additionally, bootstrapping techniques were employed to minimize the influence of outliers and variance by creating and analyzing multiple ‘bootstrap’ datasets. Our analysis also included sensitivity and specificity assessments, providing insights into the model’s ability to accurately identify positive and negative instances. Furthermore, we conducted receiver operating characteristic (ROC) curve analysis to assess the diagnostic capability of the biomarker at various threshold levels, complemented by area under the curve (AUC) analysis for quantifying the model’s overall performance. We also adjusted for potential confounding variables to ensure the validity of our associations. This robust methodological framework aimed to enhance the reliability and validity of our findings, offering a transparent and thorough evaluation of the model’s performance.

## 3. Results

### 3.1. High Sensitivity and Specificity in Cytokine Detection Using the FirePlex Immunoassay

To assess the sensitivity and specificity of the FirePlex Immunoassay for cytokine detection, we prepared serial dilutions of eight standard analyte proteins in nuclease-free water, resulting in concentrations of 0.1, 1, 10, 100, 1000, and 10,000 pg/mL. These dilutions were subsequently analyzed using the FirePlex-96 platform. The immunoassay can detect cytokines at concentrations as low as 0.5 pg/mL across a dynamic range spanning from 0.1 to 10,000 pg/mL (Supplementary Figure S1 and Supplementary Table S2). Additionally, the immunoassay exhibited a high level of specificity, as it consistently yielded positive results only for the targeted cytokine, with no instances of cross-reactivity with other cytokines (Supplementary Figure S2). To assess reproducibility and precision, the diluted samples were partitioned into three segments and tested on days 1, 14, and 30, respectively. Inter-assay precision was evaluated by determining the coefficient of variation (CV) of test samples at varied dilutions. Intra-assay precision was ascertained by calculating the CV across different wells within the same plate. The immunoassay exhibited CVs ranging from 5.5 to 11.3% for the quantification of targets, signifying its robust reproducibility and precision in cytokine detection (Supplementary Tables S3–S5).

### 3.2. Distinct Cytokine Levels in Plasma across Various Ethnic Groups

**Figure 1:**
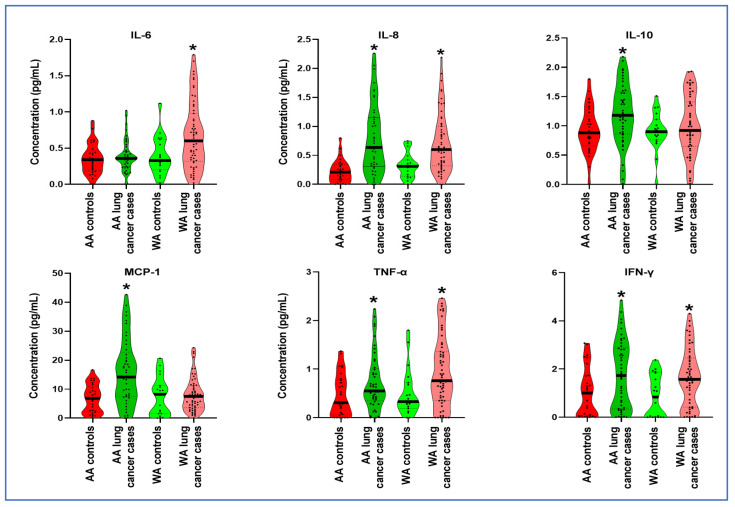
Cytokine levels in plasma of AA and WA lung cancer patients compared to their respective control groups. WA lung cancer patients exhibited significantly increased levels of IL-6 in contrast to their controls. IL-8, IFN-γ, and TNF-α displayed elevated expression levels in both AA and WA lung cancer patients compared to their respective control groups. IL-10 and MCP-1 showed exclusive upregulation in AA lung cancer cases when compared to their counterparts. *, *p* < 0.01.

IL-8, IFN-γ, and TNF-α displayed significantly elevated expression levels in both AA and WA lung cancer patients compared to their respective counterparts (all *p* < 0.05) (Table 2, Figure 1). Furthermore, in the plasma samples of AA participants, IL-10 and MCP-1 exhibited a notable increase in lung cancer patients compared to cancer-free African American controls (*p* < 0.0001) (Table 2, Figure 1). Conversely, WA lung cancer patients demonstrated significantly elevated levels of IL-6 compared to their controls (*p* = 0.017) (Table 2, Figure 1).

We utilized Spearman’s rank correlation coefficient to analyze the relationship between cytokine levels and the clinical and demographic data of lung cancer patients. Our findings indicated that increased levels of IL-6, IL-10, and MCP-1 were associated with various histological types (all *p* < 0.05) (Supplementary Table S6). Additionally, the analysis revealed that IL-10 levels were linked to patient age, and MCP-1 levels showed a correlation with patient sex (all *p*-values < 0.05) (Supplementary Table S6). However, Spearman’s rank correlation did not identify any significant correlations among the other analyzed cytokines (all *p* > 0.05) (Supplementary Table S6).

### 3.3. The Diagnostic Utility of Plasma Cytokine Biomarkers Varies with Ethnicity

**Figure 2:**
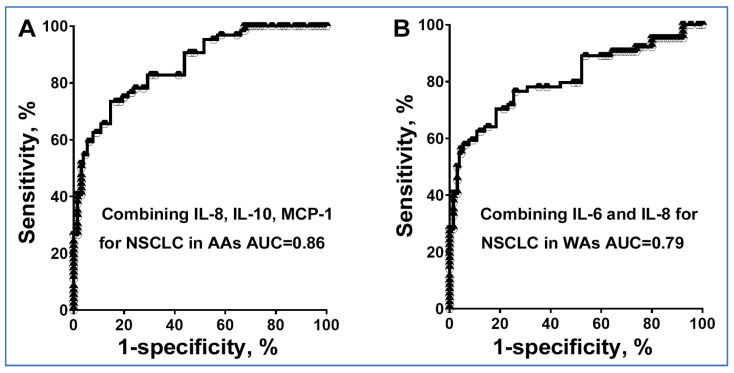
Receiver operating characteristic (ROC) curve analysis for individual cytokines and their combinations in distinguishing NSCLC patients from cancer-free smokers of different ethnicities. The area under the curve (AUC) reflects the overall diagnostic accuracy of these biomarker panels. (**A**) Combined utilization of IL-8, IL-10, and MCP-1 for the diagnosis of NSCLC in AAs. (**B**) Combined application of IL-6 and IL-8 for the diagnosis of NSCLC in WAs.

Two cytokines, IL-1 and IL-12p70, showed no significant differences between lung cancer patients and controls in both AA and WA populations. However, six other cytokines demonstrated significant variations. Additionally, our analysis found no correlations among these cytokines. To assess if combining these six cytokines could improve diagnostic accuracy for lung cancer across different ethnic groups, we utilized logistic regression and a backward elimination approach in our study. For AAs, optimal lung cancer prediction was achieved using IL-8, IL-10, and MCP-1. This panel achieved an area under the curve (AUC) of 0.86, significantly outperforming both IL-8 and MCP-1 when used individually (AUC = 0.80 or 0.76, all *p* < 0.05) to distinguish AA cancer patients from their healthy counterparts (Figure 2) (Table 3). Consequently, this biomarker panel exhibited a sensitivity of 76% and a specificity of 79% for diagnosing lung cancer in AAs (Figure 2) (Table 3). Moreover, within the AA cohort, the biomarker panel unveiled enhanced sensitivity in identifying AC as opposed to SCC (81% vs. 67%, *p* = 0.038), while maintaining a consistent specificity of 79% (Supplementary Table S7).

For WAs, the most accurate prediction was achieved using two cytokines, IL-6 and IL-8. This panel yielded an AUC of 0.80, significantly surpassing the performance of IL-6 and IL-8 when used alone (AUC = 0.70 or 0.75, all *p* < 0.05) to distinguish WA cancer patients from their healthy counterparts (Figure 2) (Table 3). As a result, this biomarker panel demonstrated a sensitivity of 76% and a specificity of 74% for the diagnosis of lung cancer in WAs (Figure 2) (Table 3). Additionally, within the WA cohort, the biomarker panel demonstrated an elevated sensitivity in identifying AC over SCC (80% vs. 71%, *p* = 0.035), whilst maintaining a steadfast specificity of 74% (Supplementary Table S7). 

When deployed collectively as panels, these biomarkers did not exhibit associations with patients’ age, sex, smoking history, pulmonary nodule size, or tumor stages of NSCLC. These findings underscore the diagnostic relevance of the plasma cytokine biomarker panel for the early detection of lung cancer in both AA and WA populations.

### 3.4. Validating the Diagnostic Potential of Cytokine Biomarkers for Detecting Disparities in Lung Cancer

We validated the cytokine biomarkers for diagnosing lung cancer within the validation cohort. Among AAs, the biomarker panel, composed of IL-8, IL-10, and MCP-1, achieved a sensitivity of 75% and a specificity of 79% for lung cancer detection (Supplementary Table S8). Furthermore, within the AA cohort, this biomarker panel demonstrated increased sensitivity in identifying AC compared to SCC (80% vs. 69%, *p* = 0.023), while maintaining a consistent specificity of 79% (Supplementary Table S8). For WAs, the panel comprising IL-6 and IL-8 exhibited a sensitivity of 77% and a specificity of 72% (Supplementary Table S7). Additionally, within the WA cohort, this biomarker panel displayed heightened sensitivity in identifying AC over SCC (82% vs. 77%, *p* = 0.019), while maintaining a consistent specificity of 72% (Supplementary Table S8).

Our primary aim in this study was to develop diagnostic markers for the early detection of lung cancer in AAs. To achieve this, we extended our validation of the initial panel of plasma cytokine biomarkers (IL-8, IL-10, and MCP-1) to include additional plasma samples from AA patients diagnosed with stage I NSCLC (Supplementary Table S1). As demonstrated in Supplementary Table S9, this biomarker panel attained a sensitivity of 75% and a specificity of 79% in detecting lung cancer in stage I NSCLC among AAs. These results are consistent with those obtained from our earlier cohort, further suggesting that cytokine biomarkers have significant potential in establishing a more effective and clinically relevant method for the early detection of lung cancer in AAs.

## 4. Discussion

Given the remarkably elevated incidence and mortality rates of lung cancer among AAs, there is an urgent need for non-invasive molecular biomarkers specifically tailored to this demographic. While previous studies have unveiled certain variations in cytokines in the blood between AA and WA patients [10,11], this domain still lacks non-invasive molecular biomarkers designed for early lung cancer detection in the AA population. It has been suggested that cytokines can influence tumor cell growth, invasion, and metastasis [11,12]. Tyan et al. have demonstrated that elevated levels of specific cytokines are associated with an increased risk of lung cancer within the AA population [11,12]. However, no plasma biomarker has yet been developed that could potentially be used for the diagnosis of lung cancer in this population. Our study represents a significant innovation compared to previous research [11,12], as it meticulously examines key cytokines in the plasma of both AA and WA lung cancer patients, contrasting them with cancer-free controls. This comprehensive analysis has revealed distinct cytokine profiles for each demographic group, leading to the development of two tailored diagnostic panels specific to each population. Notably, these panels have shown an enhanced ability to detect AC over SCC in both AA and WA groups. This is particularly crucial given that AC is the most common form of lung cancer. The ability to accurately distinguish this specific histological subtype bears substantial clinical relevance. Furthermore, none of the cytokines were associated with the stage of lung cancer. Additionally, when combined into biomarker panels, these cytokines did not show associations with different tumor stages in lung cancer patients. In particular, the diagnostic performance of the plasma cytokine biomarkers panel specifically for the early stage of lung cancer in AAs was validated in both the first and second sets of cohorts. These findings underscore the value of these novel biomarkers in advancing personalized medicine, offering significant implications for early and tailored cancer interventions.

In both AA and WA lung cancer patients, IL-8, IFN-γ, and TNF-α showed higher levels in plasma samples compared to the cancer-free controls. IL-8 is produced by various cells and plays multiple roles in lung cancer, including the promotion of crucial angiogenesis for tumor growth and facilitating tumor cell proliferation, migration, and invasion [18]. Additionally, elevated IL-8 levels have been correlated with resistance to EGFR inhibitors and poorer patient outcomes of lung cancer, highlighting its potential as both a therapeutic target and a prognostic biomarker [19]. IFN-γ is a key player in promoting anti-tumor immune responses due to its cytostatic, pro-apoptotic, and anti-proliferative properties. It also has potential in inhibiting angiogenesis, inducing regulatory T-cell apoptosis, and enhancing M1 macrophage activity to combat tumor progression [20]. TNF-α has an important role in inflammation and its involvement in inflammation-driven cancer. While it can induce cell death in some cancer types and has been used in cancer treatment, it is also implicated as an oncogenic factor in various cancers, including NSCLC. TNF activates the NF-κB transcription factor, which is a crucial mediator in inflammation-induced cancer [21]. IL-10 and MCP-1 exhibit notably elevated levels in the plasma of AA lung cancer patients. IL-10, an acid-sensitive homodimeric cytokine, is recognized as a significant immunosuppressive and pro-tumoral factor. This pleiotropic molecule exhibits anti-inflammatory properties, modulating autoimmunity, cell proliferation, survival, apoptosis, and angiogenesis [22]. It is primarily secreted by M2 macrophages, regulatory T cells, and Th2 cells. Furthermore, bronchial epithelial cells, the primary source of NSCLC, are also capable of producing IL-10 [23]. MCP-1 was significantly elevated compared to controls. MCP-1 has been implicated in lung cancer progression by recruiting tumor-associated macrophages, which foster tumor growth and metastasis, creating an immunosuppressive tumor environment and assisting tumor evasion from immune scrutiny [24]. Conversely, WA lung cancer patients exhibited significantly elevated levels of IL-6 compared to controls. The dysregulation of IL-6 stimulates tumor growth and progression and also establishes a pro-tumorigenic microenvironment [25]. Elevated IL-6 levels have been connected with advanced disease stages, poorer prognosis, resistance to specific therapies, and cancer-associated cachexia in advanced lung cancer patients [26]. Nonetheless, these cytokines’ specific roles and implications require further investigation to fully understand racial disparities in lung cancer incidence.

This study presents certain limitations, notably a modest sample size and the reliance on existing, retrospective sample sets, potentially leading to selection bias and overfitting in the development of biomarkers. To address this, our ongoing research aims to validate the plasma cytokine biomarker in a large and diverse cohort, encompassing both cases and controls. This prospective cohort, encompassing a wide array of health conditions and lifestyle factors, will facilitate a more thorough evaluation of the biomarkers’ predictive accuracy through an expanded sample collection. Additionally, our current cytokine biomarker panels, which are based on a limited selection of eight cytokines, might not yield the desired diagnostic significance in clinical settings. Given that there are approximately 80 cytokines involved in inflammatory and immune responses [27], our future research will focus on incorporating additional cytokines to enhance the efficacy of our existing biomarkers, ultimately aiming for more accurate lung cancer diagnosis. 

To translate our biomarkers into practical applications, we will optimize them for simple blood tests that are easily administered in standard clinical labs, with rapid results enabling swift clinical decisions. We will evaluate their performance in distinguishing between benign and malignant lung conditions, thus reducing unnecessary invasive procedures, healthcare costs, and patient discomfort. Additionally, we will assess the biomarkers’ significance in indicating tumor response to treatment, aiding clinicians in adjusting treatment plans. The biomarkers’ role in personalized medicine will also be explored, with clinicians using individual cytokine profiles to tailor treatments, potentially enhancing efficacy and minimizing adverse effects. Finally, we plan to integrate these biomarkers into risk stratification models, helping identify high-risk patients for early lung cancer intervention and thereby potentially improving patient outcomes.

## 5. Conclusions

The distinctive cytokine profiles linked with lung cancer in AAs as compared to WAs could serve as pivotal biomarkers in addressing the significant racial disparity in lung cancer incidence and outcomes. However, a comprehensive multi-center clinical trial is essential to confirm the true efficacy of these biomarkers for the early detection of lung cancer across diverse populations. Moreover, our research will further investigate the functional roles and biological mechanisms of these cytokines in the development of lung cancer within the AA population. This will not only provide a more comprehensive understanding of the disease process but also potentially unveil novel therapeutic targets.

## Figures and Tables

**Table 1:** Characteristics of NSCLC patients and cancer-free smokers.

An exploratory Set			A Validation Set		
	Cancer cases (*n* = 104)	Controls (*n* = 48)		Cancer cases (*n* = 58)	Controls (*n* = 58)
Age	66.62 (SD 11.58)	64.23 (SD 10.14)	Age	65.68 (SD 10.93)	65.43 (SD 10.27)
Sex			Sex		
Female	14	5	Female	12	12
Male	90	43	Male	46	46
Race			Race		
African Americans	45	29	African Americans	28	28
White Americans	59	19	White Americans	30	30
Smoking pack-years	32.7	30.3	Smoking pack-years	36.4	33.9
Stage			Stage		
Stage I	28		Stage I	18	
Stage II	16		Stage II	16	
Stage III	26		Stage III	14	
Stage IV	34		Stage IV	10	
Histological type			Histological type		
Adenocarcinoma	52		Adenocarcinoma	32	
Squamous cell carcinoma	52		Squamous cell carcinoma	26	

Abbreviations: SD, standard deviation.

**Table 2:** Mean expression levels of cytokines in AA lung cancer patients and WA lung cancer patients compared to their cancer-free counterparts.

	AA Controls	AA Cancer Patients	U Statistic	*p*	WA Controls	WA Cancer Patients	U Statistic	*p*
IL-6	0.355	0.388	589.0	0.485	0.397	0.672	357.0	0.018 *
IL-8	0.228	0.784	245.5	<0.0001 *	0.323	0.709	268.5	0.008 *
IL-10	0.917	1.195	421.5	0.011 *	0.906	0.999	440.0	0.561
MCP-1	6.848	16.080	317.0	0.001 *	7.533	8.087	510.0	0.560
IFN-γ	1.026	1.781	396.0	0.007 *	0.8748	1.659	355.0	0.002 *
TNF-α	0.470	0.739	0453.0	0.045 *	0.518	0.941	285.0	0.013 *

*, *p* < 0.05. The Shapiro–Wilk Test showed that our data were not normally distributed, and we used the Mann–Whitney U Test to analyze cytokine differences between groups.

**Table 3:** Diagnostic performance of plasma cytokines for lung cancer.

Cytokines	AUC (95% CI)	Sensitivity (%) (95% CI)	Specificity (%) (95% CI)
IL-6 in WAs	0.7035 (0.5846 to 0.8223)	71.64 (59.31% to 81.99%)	63.16 (38.36% to 83.71%)
IL-8 in WAs	0.7440 (0.6297 to 0.8583)	71.19 (57.92% to 82.24%)	68.42 (43.45% to 87.42%)
IL-8 in AAs	0.8076 (54.80% to 83.24%)	75.86 (56.46% to 89.70%)
MCP-1 in AAs	0.7571 (0.6495 to 0.8647)	68.89 (53.35% to 81.83%)	62.07 (42.26% to 79.31%)
Combined IL-6 and IL-8 in WAs	80.76 (0.7310 to 0.8842)	76.27 (63.41% to 86.38%)	73.68 (48.80% to 90.85%)
Combined IL-8, IL-10, and MCP-1 in AAs	86.15 (0.8003 to 0.9228)	75.56 (60.46% to 87.12%)	79.31 (60.28% to 92.01%)

AUC, the area under receiver operating characteristic curve; CI, confidence interval.

## Data Availability

The data that support the findings of this study are available from the corresponding author upon reasonable request.

## Supplementary Materials

The following supporting information can be downloaded at https://www.mdpi.com/article/10.3390/jpm14010117/s1. Supplementary Figure S1: The wide dynamic range assessed for eight cytokines using the FirePlex Immunoassay; Supplementary Figure S2: The analytic specificity of the FirePlex Immunoassay for cytokine detection was assessed using a panel of cytokines; Supplementary Table S1: Characteristics of Stage I NSCLC Patients and cancer-free smokers in the second validation set; Supplementary Table S2: Sensitivity and dynamic range of the FirePlex immunoassay for the detection of the eight cytokines; Supplementary Table S3: Day-to-day reproducibility of the FirePlex Immunoassay for detection of cytokines; Supplementary Table S4: Inter-assay precision of the FirePlex Immunoassay for detection of cytokines; Supplementary Table S5: Intra-assay precision of the FirePlex Immunoassay for detection of cytokines; Supplementary Table S6: Associations between the cytokines and clinical and demographic data, analyzed using Spearman’s rank correlation coefficients. *, *p* < 0.05; Supplementary Table S7: Diagnostic performance of the biomarker panels in the exploratory set; Supplementary Table S8: Diagnostic performance of the biomarker panels in AAs or Was in the validation set; Supplementary Table S9: The diagnostic values of the individual panels in the second validation set.
