# Supplementary material for: Cytokine Signatures for Lung Cancer Diagnosis in African American Populations
Source: J Pers Med. 2024 Jan 20;14(1):117. doi: 10.3390/jpm14010117 (PMC10817620; doi:10.3390/jpm14010117)
Supplement: Supplementary file 1 [file jpm-14-00117-s001.zip › jpm-2719358-supplementary.pdf]

Supplementary files

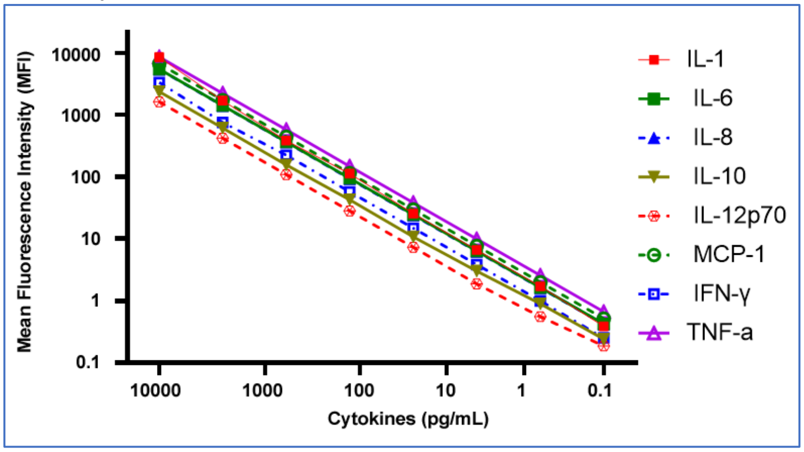

Supplementary Figure S1.

The wide dynamic range assessed for eight cytokines using the FirePlex Immunoassay. We prepared serial dilutions of standard analyte proteins in nuclease-free water, ranging from 0.1 to 10,000 pg/mL. This immunoassay reliably detects cytokines, even at 0.5 pg/mL, covering the entire 0.1 to 10,000 pg/mL range.

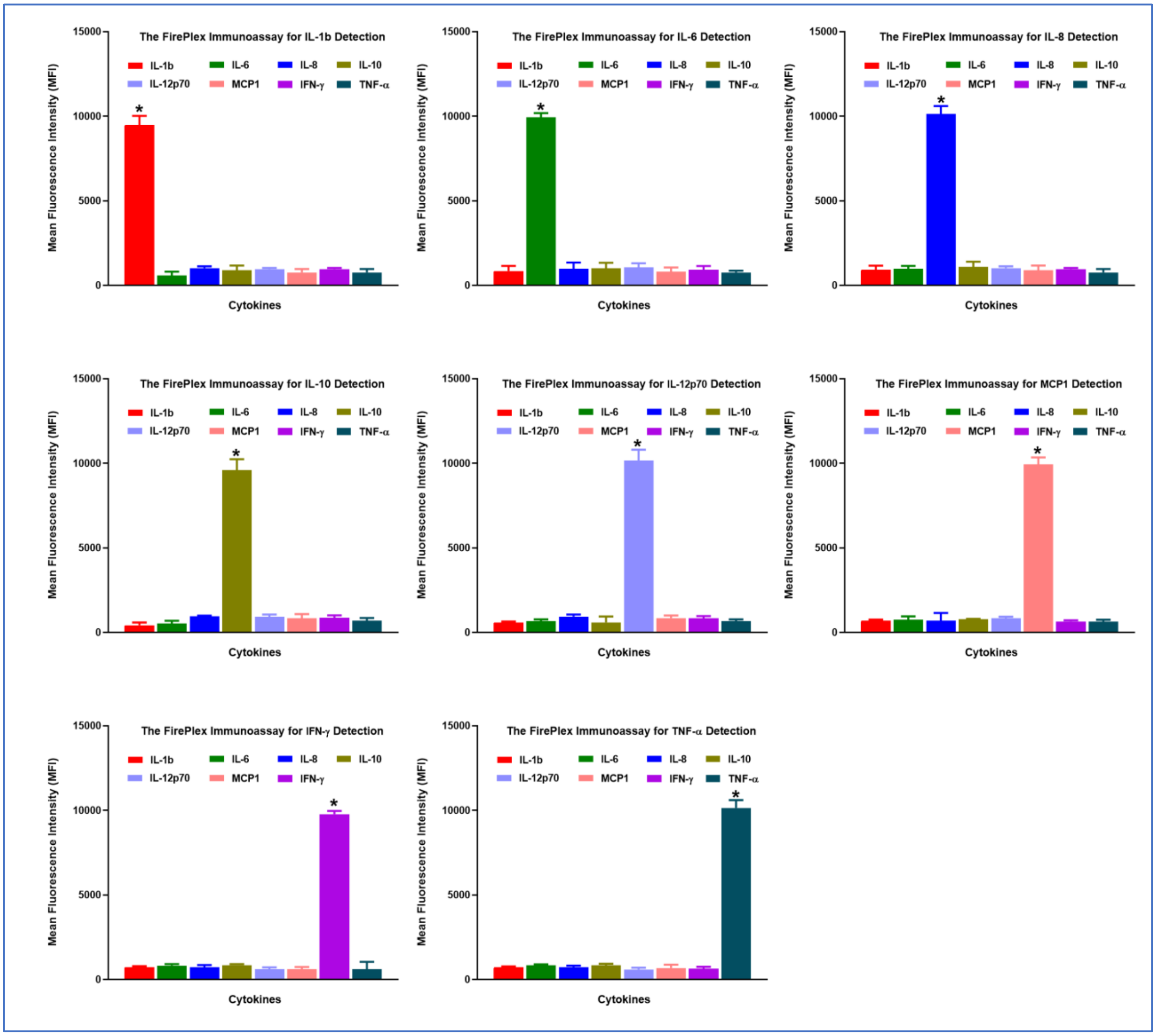

Supplementary Figure S2. The analytic specificity of the FirePlex Immunoassay for cytokine detection was assessed using a panel of cytokines. The X-axis represents each cytokine sample, while the Y-axis displays the mean fluorescence intensity (MFI) for each sample. Error bars depict the standard deviation of MFIs obtained from triplicates per sample. Significance levels are denoted as follows: * $p < 0.0001$.

Supplementary Table S1. Characteristics of Stage I NSCLC Patients and Cancer-Free Smokers in the Second Validation Set

	Cancer cases (n = 36)	Controls (n = 38)
Age	63.57<br>(SD 10.49)	65.43<br>(SD 10.46)
Sex		
Female	13	12
Male	23	26
Race	All are AAs	All are AAs
Smoking pack-years	38.3	35.7
Stage	All are Stage I	
Histological type		
Adenocarcinoma	16	
Squamous cell carcinoma	20	

Supplementary Table S2. Sensitivity and dynamic range of the FirePlex Immunoassay for the detection of the eight cytolokes.

Cytokines	Dynamic Range (pg/mL)	Sensitivity (pg/mL)
IL-1	1.62-8,970	0.79
IL-6	4.73-10,000	2.73
IL-8	0.56-9,984	0.55
IL-10	2.84-10,000	0.84
IL-12p70	4.18-10,000	1.57
MCP-1	1.95-10,000	0.72
IFN-γ	4.37-10,000	1.53
TNF-α	3.78-10,000	1.05

Supplementary Table S3. Day to day reproducibility of the FirePlex Immunoassay for the detection of cytokines.

Cytokines	*CV (%)
IL-1	9.12
IL-6	7.49
IL-8	11.59
IL-10	10.35
IL-12p70	11.17
MCP-1	5.52
IFN-γ	6.39
TNF-α	8.37
*, CV, coefficient of variation.	

Supplementary Table S4. Inter-assay precision of the FirePlex Immunoassay for the detection of cytokines.

Cytokines	*CV (%)

IL-1	11.32
IL-6	7.536
IL-8	12.67
IL-10	10.58
IL-12p70	9.53
MCP-1	11.73
IFN-γ	7.54
TNF-α	6.72
*, CV, coefficient of variation.	

Supplementary Table S5. Intra-assay precision of the FirePlex Immunoassay for the detection of cytokines.

Cytokines	CV (%)
IL-1	7.904
IL-6	10.25
IL-8	7.742
IL-10	11.43
IL-12p70	7.32
MCP-1	9.72
IFN-γ	10.21
TNF-α	6.45

Supplementary Table S6. Associations between cytokines and clinical and demographic data, analyzed using Spearman's rank correlation coefficients. *, p<0.05.

	IL-6	IL-8	IL-10	MCP1	IFN-γ	TNF-α	Age	Sex	Race	Smoking	Stage	Histology
IL-6	1.00	0.31	0.28	0.16	0.07	0.33	0.21	-0.09	0.11	0.18	0.17	**0.04***
IL-8	0.31	1.00	0.26	0.37	0.07	0.35	0.20	-0.01	0.12	0.02	0.14	-0.08
IL-10	0.28	0.26	1.00	0.25	-0.13	0.40	***0.03****	0.07	-0.08	-0.13	-0.05	***0.03****
MCP1	0.16	0.37	0.25	1.00	-0.12	0.42	-0.09	***0.02****	0.20	-0.09	0.10	***0.02****
IFN-γ	0.07	0.07	-0.13	-0.12	1.00	-0.04	-0.01	0.36	-0.02	0.11	-0.08	-0.20
TNF-α	0.33	0.35	0.40	0.42	-0.04	1.00	0.36	0.07	0.14	-0.09	-0.01	-0.19
Age	0.21	0.20	***0.03****	-0.09	-0.01	0.04	1.00	-0.13	0.29	0.36	-0.05	0.14
Sex	-0.09	-0.01	0.07	***0.02****	0.36	0.07	-0.13	1.00	-0.03	-0.13	***0.01****	-0.28
Race	0.11	0.12	-0.08	0.20	-0.02	0.14	0.29	-0.03	1.00	0.37	0.06	0.25
Smoking	0.18	0.02	-0.13	-0.09	0.11	-0.09	0.36	-0.13	0.37	1.00	0.12	0.34
Stage	0.17	0.14	-0.05	0.10	-0.08	-0.01	-0.05	***0.01****	0.06	0.12	1.00	-0.05
Histology	***0.04****	-0.08	***0.03****	***0.02****	-0.20	-0.19	0.14	-0.28	0.25	0.34	-0.05	1.00

Supplementary Table S7. The diagnostic values of the individual panels in the exploratory set.

	Sensitivity (%) (95% CI)	Specificity (%) (95% CI)
A panel of IL-8, IL-10, and MCP-1 for diagnosis of NSCLC in AAs	75.56% (60.46% to 87.12%)	79.31% (60.28% to 92.01%)
A panel of IL-8, IL-10, and MCP-1 for diagnosis of AC in AAs	81.48% (61.92% to 93.70%)	79.31% (60.28% to 92.01%)
A panel of IL-8, IL-10, and MCP-1 for diagnosis of SCC in AAs	66.67% (40.99% to 86.66%)	79.31% (60.28% to 92.01%)
A panel of IL-6 and IL-8 for diagnosis of NSCLC in WAs	76.27% (63.41% to 86.38%)	73.68% (48.80% to 90.85%)

A panel of IL-6 and IL-8 for diagnosis of AC in WAs	80.00% (63.06% to 91.56%)	73.68% (48.80% to 90.85%)
A panel of IL-6 and IL-8 for diagnosis of SCC in WAs	70.83% (48.91% to 87.38%)	73.68% (48.80% to 90.85%)

Supplementary Table S8. The diagnostic values of the individual panels in the validation set.

	Sensitivity (%) (95% CI)	Specificity (%) (95% CI)
A panel of IL-8, IL-10, and MCP-1 for diagnosis of NSCLC in AAs	75.00% (55.13% to 89.31%	79.26% (64.28% to 86.85%)
A panel of IL-8, IL-10, and MCP-1 for diagnosis of AC in AAs	80.00% (51.91% to 95.67%)	79.26% (64.28% to 86.85%)
A panel of IL-8, IL-10, and MCP-1 for diagnosis of SCC in AAs	69.23% (38.57% to 90.91%)	79.26% (64.28% to 86.85%)
A panel of IL-6 and IL-8 for diagnosis of NSCLC in WAs	76.67% (57.72% to 90.07%)	72.39% (59.10% to 83.34%)
A panel of IL-6 and IL-8 for diagnosis of AC in WAs	82.35% (56.57% to 96.20%)	72.39% (59.10% to 83.34%)
A panel of IL-6 and IL-8 for diagnosis of SCC in WAs	76.92% (46.19% to 94.96%)	72.39% (59.10% to 83.34%)

Supplementary Table S9. The diagnostic values of the individual panels in the second validation set.

	Sensitivity (%) (95% CI)	Specificity (%) (95% CI)
A panel of IL-8, IL-10, and MCP-1 for diagnosis of NSCLC in AAs	75.00% (57.80% to 87.88%)	79.95% (62.98% to 90.45%)
